# 10.H. Round table: Access, equity and the relevance of the Pandemic Treaty: lessons from the COVID-19 response

**DOI:** 10.1093/eurpub/ckac129.648

**Published:** 2022-10-25

**Authors:** Organised by: EUPHA-HTA, EUPHA-LAW, European Association of Health Law, Chair persons: Dineke Zeegers Paget (EUPHA), Elena Petelos (EUPHA-HTA)

## Abstract

Access to medicines, equity and fair pricing have been intensely debated in the context of EU policies and global health priorities for many years prior to the COVID-19 pandemic. To this effect, there have been efforts to develop mechanisms to jointly assess health technologies (HTA Regulation) and to establish cross-border partnerships for price negotiation and procurement. Although TFEU (art.168) is limiting the scope of activities and decisions to be taken at the EU level for public health matters, the Commission has also played an active part in global health discussions in the G20 and in the G7 since 2010, and there is explicit commitment to SDGs, and in particular UHC (SDG3.8) ‘leaving no one behind’ (2019). Indeed, according to its commitments “the EU advocates equitable, universal and high-quality healthcare coverage and promotes fair, effective financing of research to benefit the health of all [and] it is working to ensure that new products are safe, effective, accessible and affordable.” In the context of the COVID-19 pandemic, the EU came together in solidarity within and beyond its borders, including through joint procurement of countermeasures. At the same time, different instruments were developed to combat the pandemic globally, such ACT-Accelerator deploying the diagnostic and medicinal products, including, vaccines, and its COVAX Global Vaccines Facility to scale up production and establish consensus on the international allocation of these products. In this transitional phase, issues that arise are:

What is the role of the EU in shaping European public health and global health? What are the fundamental principles enshrined in legislative and non-legislative instruments determining EU policies and actions of global health impact? Is such EU policymaking informed by evidence? Are the necessary interdisciplinary and intersectoral partnerships in place to generate the necessary evidence?

Which instruments and mechanisms have been the most efficient and effective in combating the COVID-19 pandemic? Are they relevant for future pandemics and for increasing preparedness and resilience? What has been the role and contribution of the EU in these initiatives? What are the values and fundamental principles determining EU action and policy, and to what degree are EU policies evidence-informed? Will the EU's efforts be determined by G20 decision-making, interests and priorities?

Each panel member will briefly (5 min) present work conducted to inform European public health and global health policies. The panel will then discuss EU public health and global health policies, assessing the extent to which they are evidence-informed and the degree to which they reflect the fundamental principles of the Union. There will be two rounds of questions/statements, the first elucidating the role of the EU, the second examining it in the context of the COVID-19 pandemic and future pandemics, incl. in the context of the development of the Pandemic Treaty.

**Key messages:**

• Sound governance, transparency and implementation monitoring is required for all instruments and mechanisms to combat the COVID-19 pandemic and ones, with due consideration to sustainability and UHC.

• The EU’s role in global health needs to be strengthened via interdisciplinary and intersectoral collaboration for evidence-informed policies, and in a manner consistent with EU fundamental principles.

**Speakers/Panellists:**

**Dimitra Lingri**

National Organization for Health Care Services Provision, Athens, Greece

**Rosa Castro**

European Public Health Alliance, Brussels, Belgium

**Tom Buis**

Wemos, Amsterdam, Netherlands

**Aurelie Mahalatchimy**

UMR DICE CERIC & EAHL Interest Group on Supranational Biolaw, Aix-en-Provence, France

